# Supplementary material for: Piezo buffers mechanical stress via modulation of intracellular Ca2+ handling in the Drosophila heart
Source: Front Physiol. 2022 Sep 14;13:1003999. doi: 10.3389/fphys.2022.1003999 (PMC9515499; doi:10.3389/fphys.2022.1003999)
Supplement: Supplementary file 2 [file DataSheet1.PDF]

Supplementary Fig. 1: Piezo subcellular localisation in salivary glands

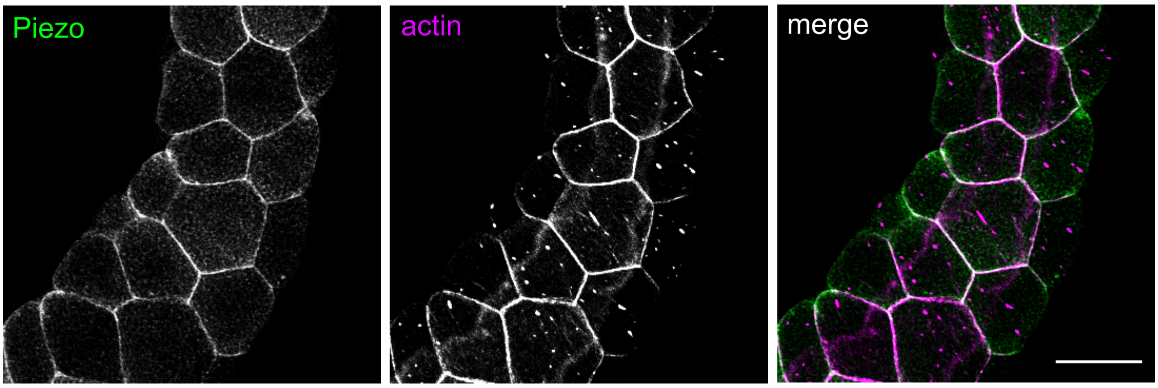

Supplementary Fig.1. Piezo subcellular localisation in *Drosophila* larval salivary gland.
Piezo-Gal4>Piezo::GFP (green) in *Drosophila* third instar larval salivary gland counterstained with phalloidin to mark cortical actin (magenta). Piezo localises predominantly to the plasma membrane. Staining for individual channels (Piezo, left; actin, middle) and merged channel (right) are shown. Scale bar = 100 μm.

Supplementary Fig. 2: *Piezo* hearts are physiologically normal

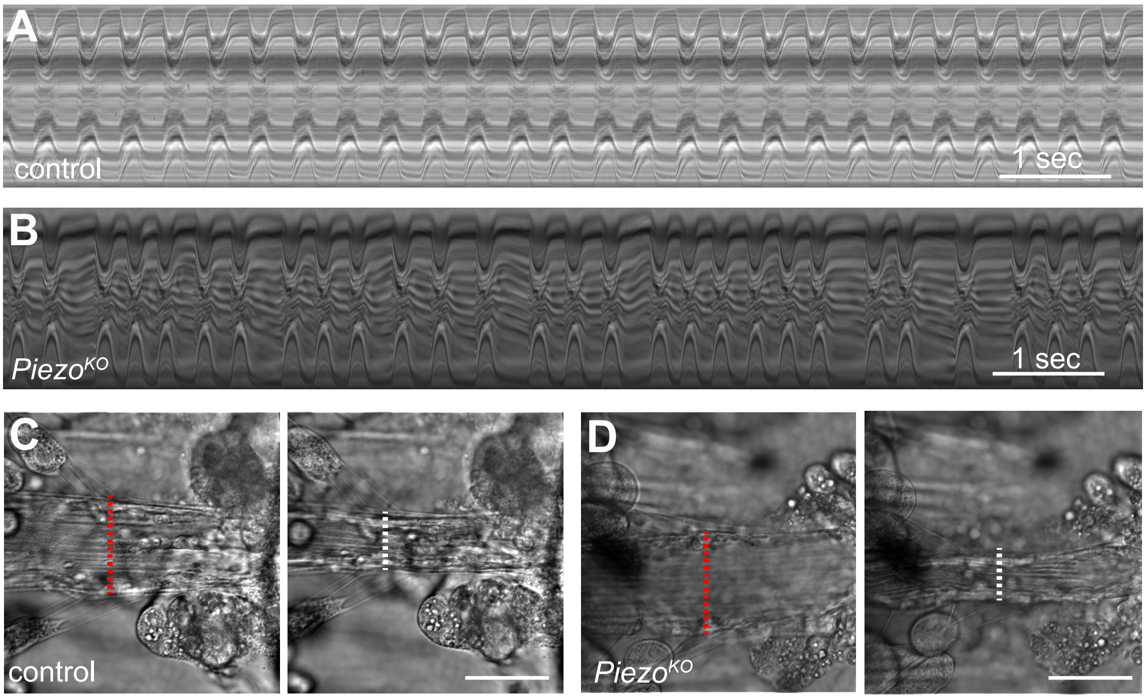

E

	control	*Piezo*^{KO}
Length of cadiac cycle (ms)	35 ± 3.3	36 ± 1.4
Beats per second	2.9	2.8
End diastolic diameter (µm)	62.5 ± 2.1	67.4 ± 2.4
End systolic diameter (µm)	37.6 ± 1.8	35.4 ± 1.4

Supplementary Fig. 2. *Piezo* hearts are physiologically normal
(A,B) M-mode kymograph traces for control (A) and *Piezo*^{KO} (B) adult hearts. (C,D) Single frame images from movies showing heart wall movement in control (C) and *Piezo*^{KO} (D) hearts in diastole (red dashed line) and systole (white dashed line). Scale bar in C,D = 50 µm. (E) Parameters of heart function for control and *Piezo*^{KO} hearts (± = S.E.M.). No significant differences are found for end diastolic diameter and end systolic diameter when comparing between control versus *Piezo*^{KO} hearts: *P*-values = 0.14 and 0.34 respectively (unpaired t-test).

Supplementary Fig. 3: Custom-made devices for modulation of ambient pressure

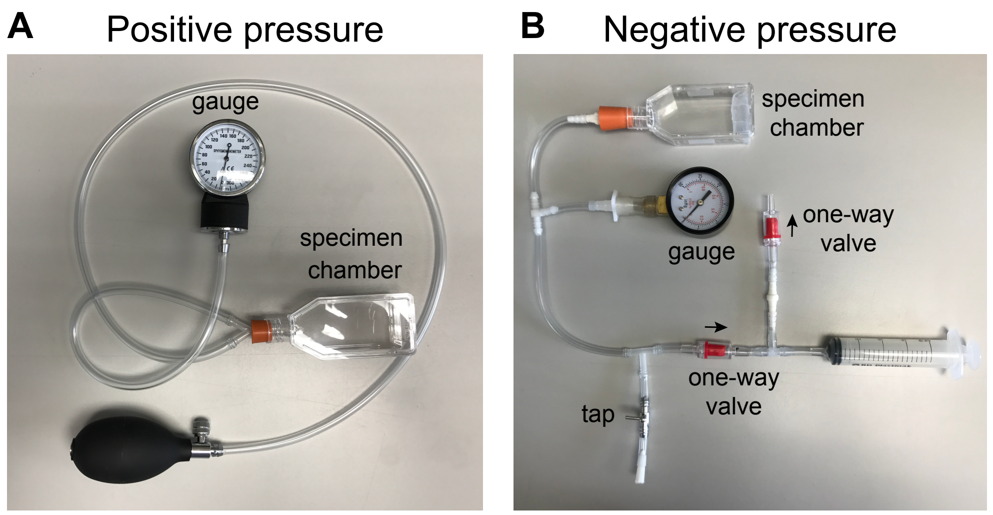

Supplementary Fig. 3. Custom-made devices for modulation of ambient pressure
(A) Positive pressure device. The specimen chamber was made from a 25cm^2 cell culture flask, plugged with a rubber bung. The specimen chamber is connected to a bulb pump and a gauge (components obtained from a sphygmomanometer) via PVC tubing. (B) Negative pressure device. The specimen chamber was made from a 25cm^2 cell culture flask, plugged with a rubber bung. The specimen chamber is connected to a syringe via PVC tubing; two one-way valves allow air to be extracted from the specimen chamber (arrows indicate direction of flow). A tap was incorporated to equilibrize pressure.

Supplementary Fig. 4: Caffeine-induced Ca^{2+} transients

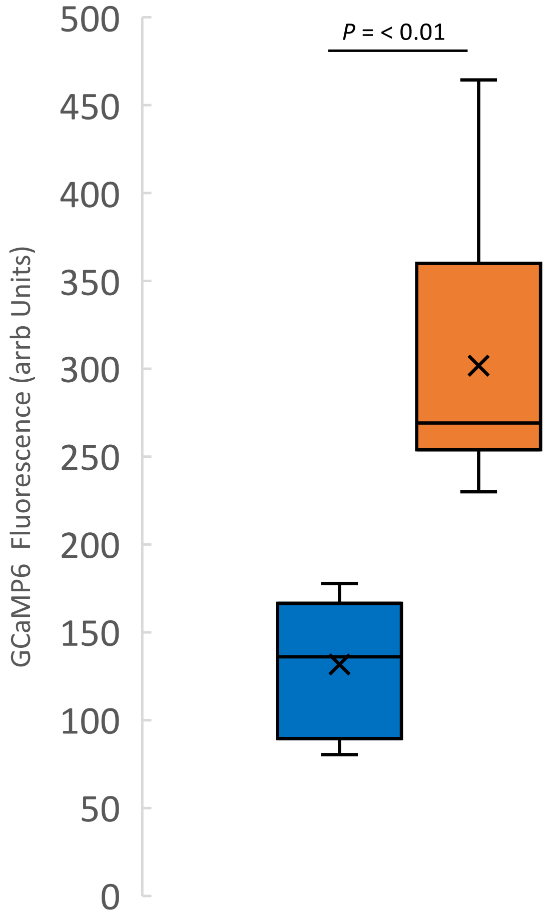

Supplementary Fig. 4. Estimation of SR Ca^{2+}content in Piezo and control cardiomyocytes

The caffeine-induced Ca^{2+} transient is significantly higher in *Piezo*^{KO} cardiomyocytes (orange) compared to controls (blue). On average the amplitude of the caffeine-induced Ca^{2+} transient in *Piezo*^{KO} cardiomyocytes is 54.3 ± 5.5% greater than that of the controls ($P<0.01$; n=7), revealing that $[Ca^{2+}]_{SR}$ is higher in *Piezo*^{KO} cardiomyocytes.
